# Comparing Native Crystal Structures and AlphaFold2 Predicted Water-Soluble G Protein-Coupled Receptor QTY Variants

**DOI:** 10.3390/life11121285

**Published:** 2021-11-24

**Authors:** Michael A. Skuhersky, Fei Tao, Rui Qing, Eva Smorodina, David Jin, Shuguang Zhang

**Affiliations:** 1 Laboratory of Molecular Architecture, Media Lab E15-391, Massachusetts Institute of Technology, 77 Massachusetts Avenue, Cambridge, MA 02139, USA; vex@mit.edu (M.A.S.); taofei@sjtu.edu.cn (F.T.); ruiqing@mit.edu (R.Q.); 2 Department of Brain and Cognitive Sciences, Massachusetts Institute of Technology, 77 Massachusetts Avenue, Cambridge, MA 02139, USA; 3 Laboratory of Food Microbial Technology, State Key Laboratory of Microbial Metabolism, School of Life Sciences and Biotechnology, Shanghai Jiaotong University, Shanghai 200240, China; 4 Faculty of Bioengineering and Bioinformatics, Lomonosov Moscow State University, 119991 Moscow, Russia; ribes.ev@gmail.com; 5 Avalon GloboCare Corp., Freehold, NJ 07728, USA; david@avalon-globocare.com

**Keywords:** chemokine receptors, membrane protein design, protein structural predictions, QTY code, water-soluble GPCRs

## Abstract

Accurate predictions of 3-dimensional protein structures by AlphaFold2 is a game-changer for biology, especially for structural biology. Here we present the studies of several native chemokine receptors including CCR5, CCR9, CXCR2 and CXCR4 determined by X-ray crystallography, and their water-soluble QTY counter parts predicted by AlphaFold2. In the native structures, there are hydrophobic amino acids leucine (L), isoleucine (I), valine (V) and phenylalanine (F) in the transmembrane helices. These hydrophobic amino acids are systematically replaced by hydrophilic amino acids glutamine (Q), threonine (T), and tyrosine (Y). Thus, the QTY variants become water-soluble. We also present the superimposed structures of native CCR10, CXCR5, CXCR7 and an olfactory receptor OR1D2 and their water-soluble QTY variants. Since the CryoEM structural determinations for the QTY variants of CCR10^QTY^ and OR1D2^QTY^ are in progress, it will be of interest to compare them when the structures become available. The superimposed structures show remarkable similarity within RMSD 1Å–2Å despite significant sequence differences (~26%–~33%). We also show the differences of hydrophobicity patches between the native GPCR and their QTY variants. Our study provides insight into the subtle differences between the hydrophobic helices and hydrophilic helices, and may further stimulate designs of water-soluble membrane proteins and other aggregated proteins.

## 1. Introduction

The recent release of the highly accurate protein prediction software suite AlphaFold2 has revolutionized protein science, especially for structural biology [1,2,3]. Likewise, a similar prediction program RoseTTAFold also made similar accurate protein structural predictions [4]. Together, they not only provided a promising solution for the long-standing difficult protein folding problem, and limited predictions of protein–protein interactions, but they also further stimulate research activities for protein design including design of water-soluble membrane proteins.

We previously designed several detergent-free transmembrane (TM) protein chemokine receptors and cytokine receptors for various uses using the conventional computing programs [5,6,7,8,9]. AlphaFold2 significantly reduces the time-consuming and expensive molecular simulation process and broadens the applications to wider research and biotech communities [1,2,3].

These chemokine receptors were chosen because they play many critical roles in health and diseases, and have been well characterized [10,11,12,13]. Chemokine receptors CCR5, CCR9, CCR10, CXCR2, CXCR4, CXCR5 and CXCR7 are also involved in various cancers initiation and metastasis, autoimmune diseases, viral infections and cytokine release syndrome, also called “cytokine storm”. Moreover, the crystal structures of CXCR4, CCR5, CCR9 and CXCR2 have been published [14,15,16,17]. allowing direct comparison with the AlphaFold2 predicted water-soluble QTY variants.

We also chose several chemokine receptors CCR10, CXCR5 and CXCR7 although their structures are not yet available, due to their importance in biology and medicine [10,11,12,13]. CCR10 and its ligands are uniquely involved in epithelial immunity, and CCR10 is expressed in subsets of innate-like T cells, which are localized to the skin during developmental processes in the thymus [18,19]. CXCR5 and its ligand CXCL13 are involved in chronic inflammation, infectious and immune responses, and autoimmune disorders [19]. CXCR7 is an atypical chemokine receptor (ACKR3) that does not activate G-protein-mediated signal transduction but recruits β-arrestins [20]. Rather, CXCR7 can heterodimerize with CXCR4 in order to modulate CXCR4 activities and can be activated by CXCL11 in malignant cells, leading to enhanced cell adhesion and migration. Elevated levels of CXCR7 expression are correlated with aggressive human prostate, breast and lung cancers, and promote growth and metastasis of various tumors [13,21,22,23].

It is intriguing and still an enigma how humans are able to detect and discriminate enormous numbers of odorants using only ~400 hundred olfactory receptors [24,25,26,27]. Despite the importance of understanding the structure and function of olfactory receptors as well as significant efforts and research funding, not a single olfactory receptor structure is known. One of the GPCRs we selected for the comparison is a human olfactory receptor (OR) OR1D2 which is a class I olfactory receptor with a narrow odorant recognition repertoire [28].

We are interested in the determinations of the CryoEM structures for the QTY variants of CCR10^QTY^ and OR1D2^QTY^. After these structures become available, it will be of great interest to directly compare their structures. For now, it is very interesting to compare the native structures predicted by AlphaFold2 and their water-soluble QTY counter parts.

## 2. Materials and Methods

### 2.1. Protein Sequence Alignments

The native GPCR structures and their QTY variants are predicted using AlphaFold2. The CCR5^QTY^ sequence is 67.05% identical to the native CCR5, CCR9^QTY^ sequence is 73.98% identical to the native CCR9, CXCR2^QTY^ is 74.44% identical to the native CXCR2, and the CXCR4^QTY^ sequence is 70.74% identical to the native CXCR4. The CCR10^QTY^ sequence is 77.64% identical to the native CCR10, CXCR5^QTY^ is 76.31% identical to the native CXCR5, and the CXCR7^QTY^ sequence is 73.67% identical to the native CXCR7, OR1D2^QTY^ sequence is 82.70% identical to the native OR1D2. The molecular weights (MW) and pI values of the proteins were calculate using the service provided by Expasy (https://web.expasy.org/compute_pi/, accessed on 21 November 2021).

### 2.2. AlphaFold2 Predictions

Structure predictions of the QTY variants were performed with the AlphaFold2 [1] software following the instructions at the website https://github.com/sokrypton/ColabFold (accessed on 21 November 2021) on 2 × 20 Intel Xeon Gold 6248 cores, 384 GB RAM, and a Nvidia Volta V100 GPU. Other AlphaFold2 predicted structures are obtained from the European Bioinformatics Institute (EBI, Hinxton, Cambridgeshire, UK, https://alphafold.ebi.ac.uk, accessed on 21 November 2021) and also available at Uniprot website https://www.uniprot.org (accessed on 21 November 2021). Please see Table 1 for details. 

Each UniProt ID from the dataset was extended with ID, entry name, description, and FASTA sequence. The data were taken from UniProt using custom Python code. The FASTA sequences were converted into their soluble versions using the QTY method (https://pss.sjtu.edu.cn/, accessed on 21 November 2021), followed by Protter (http://wlab.ethz.ch/protter/start/, accessed on 21 November 2021) 2D diagrams plotting. These steps were optimized via Python libraries for web applications such as requests and splinter.

### 2.3. Superimpose the Structures

The published X-ray crystal structures of native CCR5 (4MBS), CCR9 (5LWE), CXCR2 (6LFL) and CXCR4 (3ODU) are obtained from the Protein Data Bank (PDB), https://www.rcsb.org (accessed on 21 November 2021). AlphaFold2 predictions of three native chemokine receptors CCR10, CXCR5, CXCR7, and one olfactory receptor OR1D2, and their QTY variants are carried out using the AlphaFold2 program at https://github.com/sokrypton/ColabFold (accessed on 21 November 2021). All other native receptor protein sequences are obtained from Uniprot https://www.uniprot.org (accessed on 21 November 2021). Structures were aligned with MUSTANG [29].

### 2.4. Structure Visualization

We used two programs for structure visualization: PyMOL https://pymol.org/2/ (accessed on 21 November 2021) and UCSF Chimera https://www.rbvi.ucsf.edu/chimera/ (accessed on 21 November 2021). All superimposed models were produced via PyMOL, while Chimera was used for hydrophobicity representation.

## 3. Results and Discussions

### 3.1. Protein Sequence Alignments

**Figure 1:**
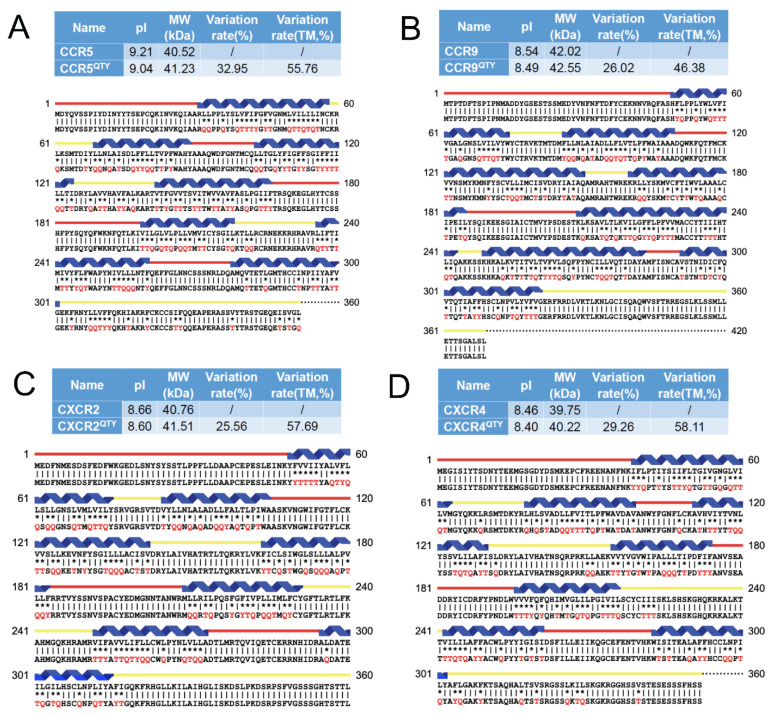
Alignment of known X-ray crystal structures of native chemokine receptors CCR5, CCR9, CXCR2 and CXCR4 with AlphaFold2 predicted native and their predicted water-soluble QTY variants. The Q, T, and Y amino acid substitutions are in red. The alpha-helical segments (blue) are shown above the protein sequences, the external (red) and internal (yellow) loops of the receptors are indicated. The symbols | and * indicate the similar and different amino acids, respectively. Characteristics of natural and QTY variants with pI, molecular weight, total variation rate and membrane variation rate, and the alignment: (**A**) CCR5 and CCR5^QTY^, (**B**) CCR9 and CCR9^QTY^, (**C**) CXCR2 and CXCR2^QTY^, and (**D**) CXCR4 and CXCR4^QTY^. Since the internal regions ICL1, ICL2, ICL3 and the C-terminus do not interact with the ligands, additional residues in these regions are sometimes QTY modified.

Since the crystal structures of native receptors CCR5, CCR9, CXCR2, and CXCR4 are already available, the sequence alignments were carried out for: CCR5 vs. CCR5^QTY^, CCR9 vs. CCR9^QTY^, CXCR2 vs. CXCR2^QTY^, CXCR4 vs. CXCR4^QTY^ (Figure 1). Thus, their crystal structures and the AlphaFold2 predicted structures can be directly compared.

The QTY code selects 3 neutral and polar amino acids without any charges: glutamine (Q), threonine (T), and tyrosine (Y) to replace hydrophobic amino acids leucine (L), isoleucine (I), valine (V), and phenylalanine (F), since their electron density maps share remarkable structure similarities [5] Supporting information). After applying the QTY code, the hydrophobic amino acids in the transmembrane segments are replaced by Q, T, and Y; therefore, the transmembrane segments are no longer hydrophobic. Although there are greater than 26% overall replacements, the protein alignments of the transmembrane α-helical segments of the native receptors and their QTY variants show similar isoelectrical point (pI) and molecular weights (Figure 1).

Although there are substantial changes, ~26–~33% overall, in the amino acid composition between the native receptors and their QTY variants, and ~46–58% in the 7TM domains, the pI changes are rather small. Respectively, being 0.06 units for CXCR2/CXCR2^QTY^, 0.06 units for CXCR4/CXCR4^QTY^, 0.05 units for CCR9/CCR9^QTY^ and 0.17 units for CCR5/CCR5^QTY^, these pI changes are insignificant regarding surface charges and unlikely to disrupt delicate structures. Likewise, the molecular weight differences are small through replacing saturated carbon side chains with −OH and −NH_2_ that can form 3–4 hydrogen bonds with water molecules. For example, CCR5/CCR5^QTY^ = 710 Daltons (1.75%), CCR9/CCR9^QTY^ = 530 Daltons (1.26%), CXCR2/CXCR2^QTY^ = 750 Daltons (1.84%), CXCR4/CXCR4^QTY^ = 470 Daltons (1.18%), these changes may be tolerated by protein structures (Figure 1).

**Figure 2:**
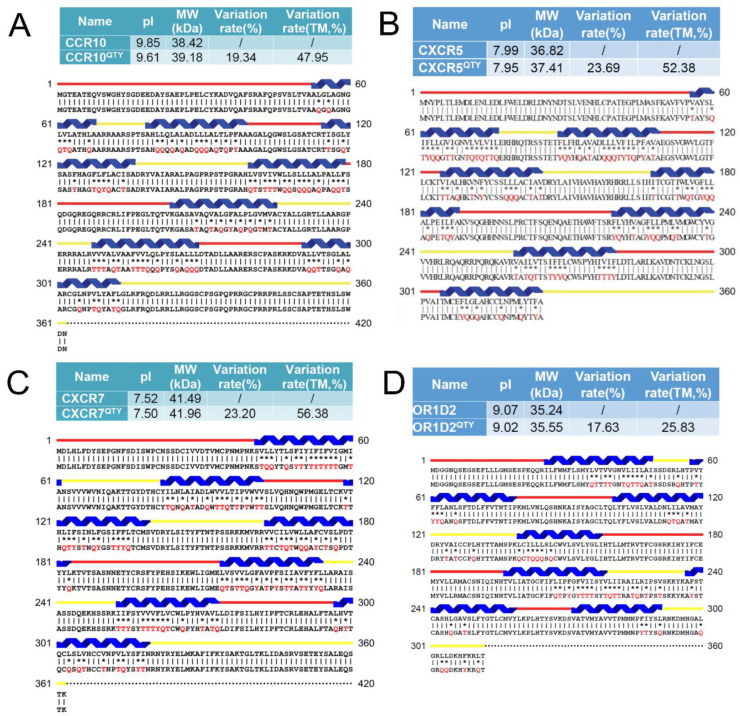
Alignment of AlphaFold2 predicted 3 native chemokine receptors and 1 olfactory receptor with their water-soluble QTY variants. The Q, T, and Y amino acid substitutions are in red. The alpha-helical segments (blue) are shown above the protein sequences, the external (red) and internal (yellow) loops of the receptors are indicated. The symbols | and * indicate the similar and different amino acids, respectively. Characteristics of natural and QTY variants with pI, molecular weight, total variation rate and membrane variation rate, and the alignment: (**A**) CCR10 and CCR10^QTY^, (**B**) CXCR5 and CXCR5^QTY^ (**C**) CXCR7 and CXCR7^QTY^ and (**D**) OR1D2 and OR1D2^QTY^. Since the internal regions ICL1, ICL2, ICL3 and the C-terminus mostly do not interact with the ligands, additional residues in these regions are sometimes also QTY modified.

We also selected several receptors that do not yet have any experimental determined structures, including CCR10, CXCR5, CXCR7 and OR1D2 and their QTY variants for alignment: CCR10 vs. CCR10^QTY^, CXCR5 vs. CXCR5^QTY^, CXCR7 vs. CXCR7^QTY^ and OR1D2 vs. OR1D2^QTY^ (Figure 2). They also have very small pI changes, i.e., 0.24 units for CCR10/CCR10^QTY^, 0.04 units for CXCR5/ CXCR5^QTY^, 0.02 units for CXCR7/CXCR7^QTY^ and 0.05 units for OR1D2/OR1D2^QTY^. Similarly, the molecular weight differences are small: CCR10/CCR10^QTY^ = 760 Daltons (1.98%), CXCR5/CXCR5^QTY^ = 590 Daltons (1.6%), CXCR7/CXCR7^QTY^ = 460 Daltons (1.26%), and OR1D2/OR1D2^QTY^ = 310 Daltons (0.82%).

### 3.2. AlphaFold2 Predictions

For over 6 decades, structural biologists and protein scientists have sought to predict how proteins fold naturally and rapidly. AlphaFold2′s accurate predictions of 3-dimensional protein structures have made such predictions a reality, revolutionizing structural biology and protein science. Such an advancement not only allows us to study protein structure more in detail but also facilitate to obtain previously unattainable protein structures through AlphaFold2 predictions, at least in the framework.

It is estimated that ~25–30% genes code for membrane proteins in most organisms [30]. However, even the determination of a single membrane protein is a dauting task, and there are many hurdles along the way, from gene expression, protein production, detergent selection, purification, to maintaining the stability, integrity, and function to avoid aggregation. Thus, despite significant advances in tools and method developments, determinations of membrane protein structures still lag far behind of those of water-soluble proteins.

The advent of AlphaFold2 has completely changed this. Using AlphaFold2 predictions, DeepMind AlphaFold’s team has already deposited over 100 million protein structures in the European Bioinformatics Institute (EBI, Hinxton, Cambridgeshire, UK, https://alphafold.ebi.ac.uk, accessed on 21 November 2021). The number will continuously increase over time.

We used the AlphaFold2 accurate prediction tool to predict water-soluble G protein-coupled receptors in this study and compare them to the known experimentally determined structures. The speed and accuracy of AlphaFold2 predictions of our designed receptors is unprecedented. Instead of taking weeks or days to predict one structure, AlphaFold2 can predict new structure in a few hours, or even minutes for smaller and simpler proteins. AlphaFold2 significantly accelerates studies of protein structures, stabilities, design new proteins, discovery of new protein interactions, and perhaps new functions that were previously unknown through experimental studies. It is a game changer for science, especially for protein science.

### 3.3. Superimpositon of Native and Water-Soluble QTY Variants

**Figure 3:**
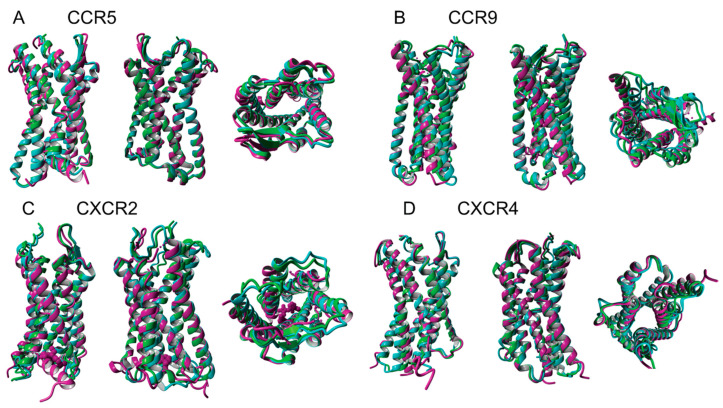
Superimposed determined 4 crystal structures, AlphaFold2 predicted 4 native chemokine receptors CCR5, CCR9, CXCR2, and CXCR4 and their QTY water-soluble variants. For each superimposition, 3 structures are show, front (left), back (middle) and view from top axis (right). The X-ray crystal structures of natural CCR5 (4MBS), CCR9 (5LWE), CXCR2 (6LFL), and CXCR4 (3ODU) are obtained from the Protein Data Bank (PDB). For clarity, the inserts for crystallization and N- and C-termini are removed. The crystal structure native (**A**) CCR5 (magenta) is superimposed with AlphaFold2 predicted native CCR5 (green) and water-soluble variant CCR5^QTY^ (cyan). Following the same order as CCR5, the superimposed determined and AlphaFold2 predicted structures of (**B**) CCR9, (**C**) CXCR2, (**D**) CXCR4.

The structures of native and water-soluble QTY variants superimposed very well except the loops and N- and C-termini. For simplicity and clarity, less-predictable N- and C-termini are not included in our predicted structures, only the 7TMs are included in the superimpositions (Figure 3). In the first sets of structures for CCR5, CCR9, CXCR2 and CXCR4, they are superimposed among: (i) the X-ray crystal determined structures CCR5 (Protein data bank code: 4MBS), CCR9 (5LWE), CXCR2 (6LFL) and CXCR4 (3ODU) (magenta color), (ii) the AlphaFold2 predicted native structures (green) and the (iii) AlphaFold2 predicted water-soluble QTY variants (cyan). As seen from the Figure 3, the structures are viewed from front, back and top, all 4 sets of structures superimposed well. The results suggest that these structures share very similar folds despite of significant QTY amino acids replacement in the water soluble QTY receptor variants. These superimpositions also suggest that the AlphaFold2′s accurate prediction capabilities since the predicted structures are directly superimposable with the experimentally determined X-ray crystal structures.

**Figure 4:**
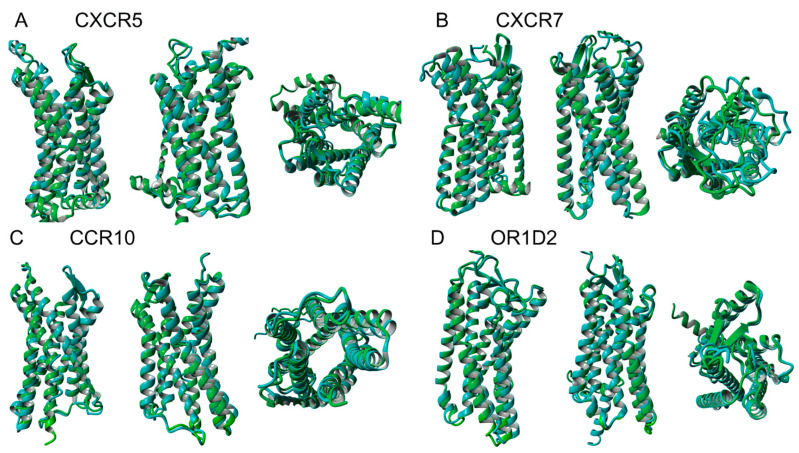
Superimposed AlphaFold2 predicted 3 native chemokine receptors and 1 olfactory receptor and their water-soluble QTY variants. For each superimposition, 3 structures are show, front (**left**), back (**middle**), and view from top axis (**right**). For clarity, N- and C-termini and large intracellular and extracellular loops are removed. (**A**) CCR10 vs. CCR10^QTY^ (RMSD 1.246 Å, 77% identity), (**B**) CXCR5 vs. CXCR5^QTY^ (RMSD 1.110 Å, 71% identity), (**C**) CXCR7 vs. CXCR7^QTY^ (RMSD 1.388 Å, 67.5% identity), and (**D**) OR1D2 vs. OR1D2^QTY^ (RMSD 0.978 Å, 83% identity).

Work is currently ongoing to confirm the QTY variant structures by CryoEM. We also directly superimposed the experimentally unknown but AlphaFold2-predicted structures of 4 native receptors and water-soluble QTY variants of chemokine receptors CCR10, CXCR5, CXCR7 and an olfactory receptor OR1D2 (Figure 4). Despite the significant differences of: (i) amino acid compositions, (ii) chemical characteristics, and (iii) sequence identities between ~67.5–83%, the structural similarity of the native and QTY variant receptors are presented by root mean square deviation (RSMD): (i) CCR10 vs. CCR10^QTY^ (RMSD 1.246 Å, 77% identity), (ii) CXCR5 vs. CXCR5^QTY^ (RMSD 1.110 Å, 71% identity), (iii) CXCR7 vs. CXCR7^QTY^ (RMSD 1.388 Å, 67.5% identity), and (iv) OR1D2 vs. OR1D2^QTY^ (RMSD 0.978 Å, 83% identity). These very closely superimposed structures again show the remarkable similarity of native and water-soluble QTY variants. This demonstrates that these alpha-helices have very similar molecular structures regardless of the hydrophobicity and hydrophilicity, namely: (i) 1.5 Å per amino acid rise, (ii) 100˚ per amino acid turn, (iii) 5.4 Å, 360˚ and 3.6 amino acids per helical turn [31,32,33].

We also carried out AlphaFold2 predictions of other variations. For example, we changed the QTY to alanine for CXCR4^QTY^, arginine or glycine for CCR5^QTY^. These results are shown in Supplementary Material Figure S1. Since alanine and arginine have propensity to form alpha-helix, the overall deviation is less than the structure change with glycine. This is not surprising, since glycine is an achiral amino acid that is very flexible and can influence the structures.

### 3.4. Analysis of the Hydrophobic Surface of Native and Water-Soluble QTY Variants

**Figure 5:**
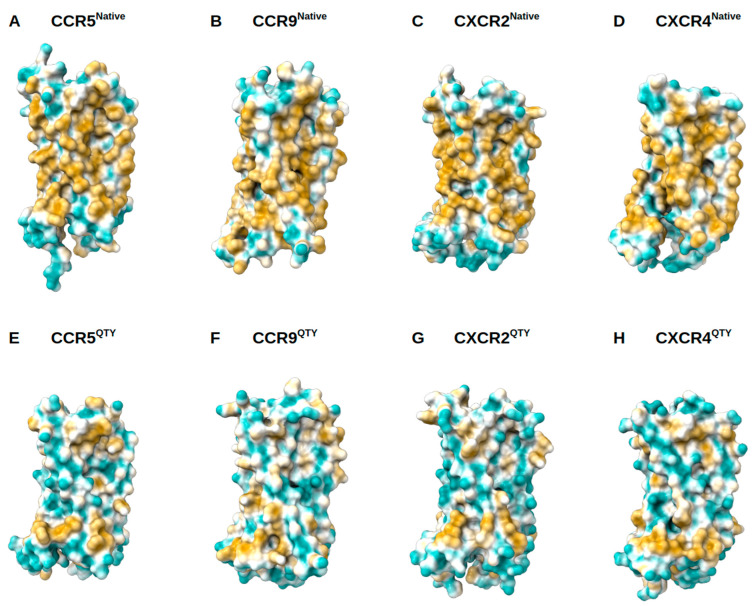
Surface hydrophobic patch of X-ray determined structures of native chemokine receptors and AlphaFold2 predicted water-soluble QTY variants. The native GPCR receptors mostly expose hydrophobic residues leucine (L), isoleucine (I), valine (V) and phenylalanine (F) facing outside to the hydrophobic lipid bilayer in cell membrane. After replacing the L, I, V, F with polar amino acids, glutamine (Q), threonine (T), and tyrosine (Y), the surfaces are much less hydrophobic. The large surface hydrophobic patch (yellow color) of the native receptors determined by X-ray crystal structures: (**A**) CCR5, (**B**) CCR9, (**C**) CXCR2 and (**D**) CXCR4. The hydrophobic patch is significantly reduced on the transmembrane domains for the AlphaFold2 predicted water-soluble QTY variants: (**E**) CCR5^QTY^, (**F**) CCR9^QTY^, (**G**) CXCR2^QTY^, (**H**) CXCR4^QTY^. These QTY variants become water-soluble without any detergent. The N- and C-termini are removed for clarity of directly comparisons.

**Figure 6:**
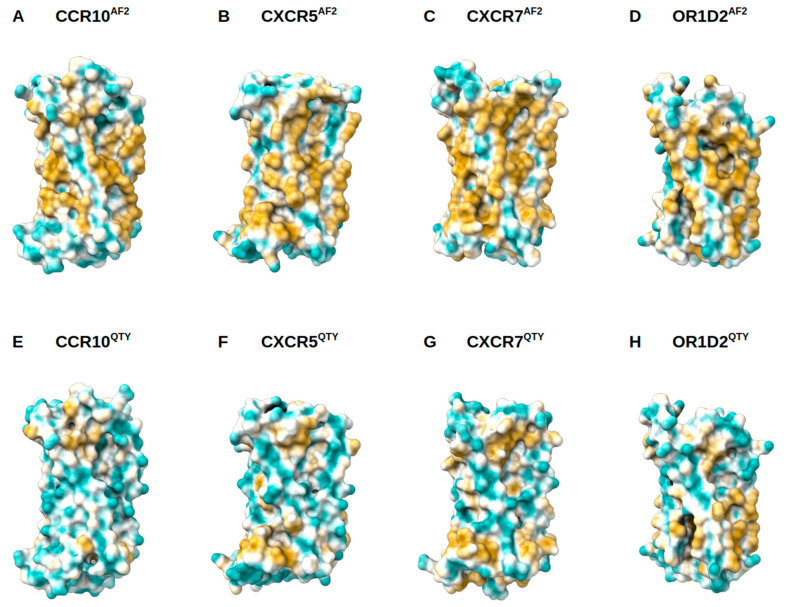
Surface hydrophobic patch of AlphaFold2 predicted structures of native chemokine receptors CCR10, CXCR5, CXCR7, and OR1D2 and their water-soluble QTY variants. The AlphaFold2 predicted structure receptors CCR10, CXCR5, CXCR7, and OR1D2 are presented. The large surface hydrophobic patch (yellow color) of the native receptors determined by AlphaFold2 predicted structures (cyan color): (**A**) CCR10, (**B**) CXCR5, (**C**) CXCR7, and (**D**) OR1D2. The hydrophobic patch is significantly reduced on the transmembrane domains for the AlphaFold2 predicted water-soluble QTY variants: (**E**) CCR10^QTY^, (**F**) CCXC5^QTY^, (**G**) CXCR7^QTY^, and (**H**) OR1D2^QTY^. These QTY variants become water-soluble without any detergent. The N- and C-termini are removed for clarity of directly comparisons.

Native GPCRs are highly hydrophobic, especially in the 7TM helical domains, thus intrinsically water-insoluble, and require detergents to solubilize them after isolating them from the lipid bilayer membranes. Without the appropriate detergents, they immediately aggregate, precipitate, and lose their biological functions. The 7TM domains are directly embedded in the hydrophobic lipid bilayer so the hydrophobic side chains of amino acids leucine (L), isoleucine (I), valine (V), and phenylalanine (F) directly interact with the lipid molecules excluding water. Thus, they display highly hydrophobic patches on the 7TM domain (Figure 5 and Figure 6).

On the other hand, after systematic QTY replacement of hydrophobic amino acids L, I, V, F, with hydrophilic amino acids glutamine (Q), threonine (T), and tyrosine (Y), these hydrophobic patches become largely reduced (Figure 5 and Figure 6). Such transformation from hydrophobic 7TM to hydrophilic 7TM did not significantly alter the alpha-helix structures, stability or their ligand binding functions (5–9). This was rather unexpected before our previously systematic experiments were carried out. However, the experimental evidence conclusively demonstrated preservation of both structure and function (5–9).

We note that nature has already made such alpha-helices in both hydrophilic form, such as those found in hemoglobin and many other water-soluble enzymes, and hydrophobic helices, such as in GPCRs and other membrane proteins. These helices have very similar molecular structures irrespective of the hydrophobicity and hydrophilicity [31,32,33].

## 4. Conclusions

Our study of systematically comparing experimentally determined structures with AlphaFold2 predicted water-soluble membrane receptors provides insight into the subtle differences between the hydrophobic helices and hydrophilic helices. They not only show the QTY code’s simplicity to directly replace hydrophobic amino acid residues in the membrane proteins while maintaining their structures, but also demonstrate the validity of the QTY code. Thus, applying the QTY code may further stimulate designs of water-soluble membrane proteins and other aggregated proteins.

## Figures and Tables

**Table 1:** AlphaFold 2 structure prediction parameters.

Parameter	Value
homooligomer	1
msa_method	mmseqs2
msa_format	fas
pair_mode	unpaired
pair_cov	50
pair_qid	20
rank_by	pLDDT
use_turbo	True
max_msa	512:1024
show_images	True
num_models	5
use_ptm	True
num_ensemble	1
max_recycles	3
tol	0
num_samples	1
subsample_msa	True
num_relax	None

## Data Availability

The AlphaFold2 predicted protein structures are at European Bioinformatics Institute (EBI, Hinxton, Cambridgeshire, UK, https://alphafold.ebi.ac.uk, accessed on 21 November 2021). The QTY code designed water-soluble variants are published in this paper.

## Supplementary Materials

The following are available online at https://www.mdpi.com/article/10.3390/life11121285/s1, supplementary materials including Figure S1, Superimposed AlphaFold2 predicted structures of CXCR4^QTY^, CXCR4^Ala^ and CCR5^QTY^ with CCR5^Arg^ and CCR5^Gly^, Figure S2, The AlphaFold2 predicted 5 structures of each GPCR, and Figure S3, Five AlphaFold2 predicted models of CCR5^QTY^.
