# Supplementary material for: Comparing Native Crystal Structures and AlphaFold2 Predicted Water-Soluble G Protein-Coupled Receptor QTY Variants
Source: Life (Basel). 2021 Nov 24;11(12):1285. doi: 10.3390/life11121285 (PMC8704054; doi:10.3390/life11121285)

Supplementary Materials

We carried out additional AlphaFold2 prediction of CXCR4^{QTY} where we replaced all QTY amino acids to alanine, as people have been using the "Alanine Scan" for generating and studying mutations. We name the new variant CXCR4^{Ala}. This CXCR4^{Ala} was subjected to AlphaFold2 prediction and the structure is then superimposed with the CXCR4^{QTY}. Since Alanine often found in alpha-helical structures, it is not surprising the structures have little change. Likewise, we also use AlphaFold2 prediction for **i**) CCR5^{Arg} and **ii**) CCR5^{Gly} and superimposed CCR5^{QTY} with **i**) CCR5^{Arg} and **ii**) CCR5^{Gly}. The superimposed structures and the RMSDs are shown in Figure S1. CXCR4^{QTY} and CXCR4^{Ala}

A) Protein sequence of CXCR4^{QTY}

MEGISIYTSDNYTEEMGSGDYDSMKEPCFREENANFNKTYQPTTYSTTYQTGTTGQGQTTQTMGYQKKQRSMTDKY
RQHQSTADQQYTTTQPYWATDATANWYFGNFQCKATHTTYTTQQYSSTQTQAYTSQDRYLAIVHATNSQRPRKQQA
EKTTYTGTWTPAQQQTTPDYTYANVSEADDRYICDRFYPNDLWTTTYQYQHTMTGQTQPGTTTQSCYCTTTSKLSHS
KGHQKRKALKTTTTQTQAYYACWQPYYTGTSTDSFILLEIIKQGCEFENTVHKWTSTTEAQAYYHCCQQPTQYAYQG
AKYKTSAQHAQTSTSRGSSQKTQSKGKRGGHSSTSTESESSSFHSS

B) Protein sequence of CXCR4^{Ala}

MEGISIYTSDNYTEEMGSGDYDSMKEPCFREENANFNKIFLPTAYSAAAATGAAGNGAAAAAMGYQKKLRSMTDKYR
AHASAADAAAAATAPAWAADAVANWYFGNFLCKAAHAAYTANAYSSAAAAAAASADRYLAIVHATNSQRPRKLLAEK
AAYAGAWAPAAAATAPDAAAANVSEADDRYICDRFYPNDLWVVVAQAQHAMAGAAAPGAAAASCYCAIISKLSHSKG
HQKRKALKTTAAAAAAAAACWAPYYAGASADSAILLEIIKQGCEFENTVHKWASATEAAAAAHCCANPAAYAAAGAKF
KTSAQHALTSVSRGSSLKILSKGKRGGHSSVSTESESSSFHSS

C) Native Protein sequence of CCR5

MDYQVSSPIYDINYYTSEPCQKINVKQIAARLLPPLYSLVFIFGFVGNMLVILILINCKRLKSMTDIYLLNLAISDLFFLLTVP
FWAHYAAAQWDFGNTMCQLLTGLYFIGFFSGIFFIILLTIDRYLAVVHAVFALKARTVTFGVVTSVITWVVAVFASLPGIIF
TRSQKEGLHYTCSSHFPYSQYQFWKNFQTLKIVILGLVLPLLVMVICYSGILKTLLRCRNEKKRHRAVRLIFTIMIVYFLF
WAPYNIVLLLNTFQEFFGLNNCSSSNRLDQAMQVTETLGMTHCCINPIIYAFVGEKFRNYLLVFFQKHIAKRFCKCCSIF
QQEAPERASSVYTRSTGEQEISVGL

D) Protein sequence of CCR5^{QTY}

MDYQVSSPIYDINYYTSEPCQKINVKQIAARLLPPQYSQTYTYGYTGNMQTTQTQTNCKRLKSMTDIYQQNQATSDQY
YQQTTPYWAHYAAAQWDFGNTMCQLLTGQYYTGYYSGTYYTTQQTTDRYLAVVHAVFALKARTVTYGTTTSTTTWT
TATYASQPGTTYTRSQKEGLHYTCSSHFPYSQYQFWKNFQTLKITTQGQTQPQQTMTTCYSGTQKTQLRCRNEKKR
HRATRQTYTTMTTYYQYWAPYNTTQQLNTFQEFFGLNNCSSSNRLDQAMQVTETLGMTHCCINPIIYAFVGEKFRNYL
LVFFQKHIAKRFCKCCSIFQQEAPERASSVYTRSTGEQEISVGL

E) Protein sequence of CCR5^{Arg}

MDYQVSSPIYDINYYTSEPCQKINVKQIAARLLPPRYSRRRRRGRRGNMRRRRRRRNCKRLKSMTDIYRRNRARSDR
RRRRTRPRWAHYAAAQWDFGNTMCQLLTGRYRRGRRSGRRRRRRRTRDRYLAVVHAVFALKARTVTRGRRTSRRT
WRRARRASRPGRRRTRSQKEGLHYTCSSHFPYSQYQFWKNFQTLKIRRRGRRRPRRRMRRCYSGRRKTRLRCRNE
KKRHRARRRRRTRMRRYRRRWAPYNRRRRLNTFQEFFGLNNCSSSNRLDQAMQVTETLGMTHCCINPIIYAFVGEKF
RNYLLVFFQKHIAKRFCKCCSIFQQEAPERASSVYTRSTGEQEISVGL

F) Protein sequence of CCR5^{Gly}

MDYQVSSPIYDINYYTSEPCQKINVKQIAARLLPPGYSGGGGGGGGGNMGGGGGGGNCKRLKSMTDIYGGNGAGSD
GGGGGTGPGWAHYAAAQWDFGNTMCQLLTGGYGGGGGSGGGGGGGGTGDRYLAVVHAVFALKARTVTGGGGTS
GGTWGGAGGASGPGGGGTRSQKEGLHYTCSSHFPYSQYQFWKNFQTLKIGGGGGGGPGGGMGGCYSGGGKTGL
RCRNEKKRHRAGRGGGTGMGGYGGGWAPYNGGGGLNTFQEFFGLNNCSSSNRLDQAMQVTETLGMTHCCINPIIY
AFVGEKFRNYLLVFFQKHIAKRFCKCCSIFQQEAPERASSVYTRSTGEQEISVGL

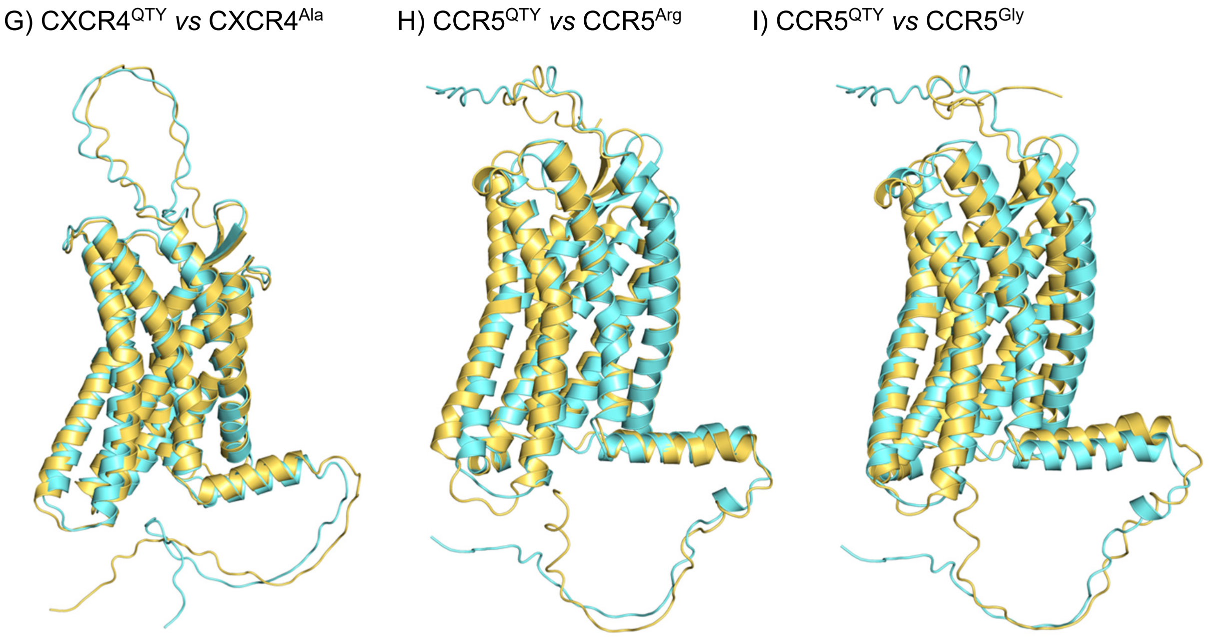

Figure S1. Superimposed AlphaFold2 predicted structures of CXCR4^{QTY}, CXCR4^{Ala} and CCR5^{QTY} with CCR5^{Arg} and CCR5^{Gly}. The protein structures are predicted using AlphaFold2, A) Protein sequence of CXCR4^{QTY}, B) Protein sequence of CXCR4^{Ala}. C) Protein sequence of CCR5^{QTY}. The superimposed structures of CXCR4^{QTY} (cyan color) and CXCR4^{Ala} (yellow color), G) superimposed CXCR4^{QTY} and CXCR4^{Ala}, H) superimposed CCR5^{QTY} and CCR5^{Arg} (RMSD = 5.140), I) superimposed CCR5^{QTY} and CCR5^{Gly} (RMSD = 6.169). The loops, N-and C-termini predictions are less accurate. Since Alanine are often found in alpha-helical structures, it is not surprising the structures have little change. On the other hand, glycine sometime destabilizes alpha-helical structures, the superimposed structures have bigger deviations.

We used the AlphaFold to predict 5 structures. Since the 5 predicted structures are similar, for clarity, we only selected one to superimpose with the crystal structure. The 5 AlphaFold to predicted 5 structures are shown in Figure S2.

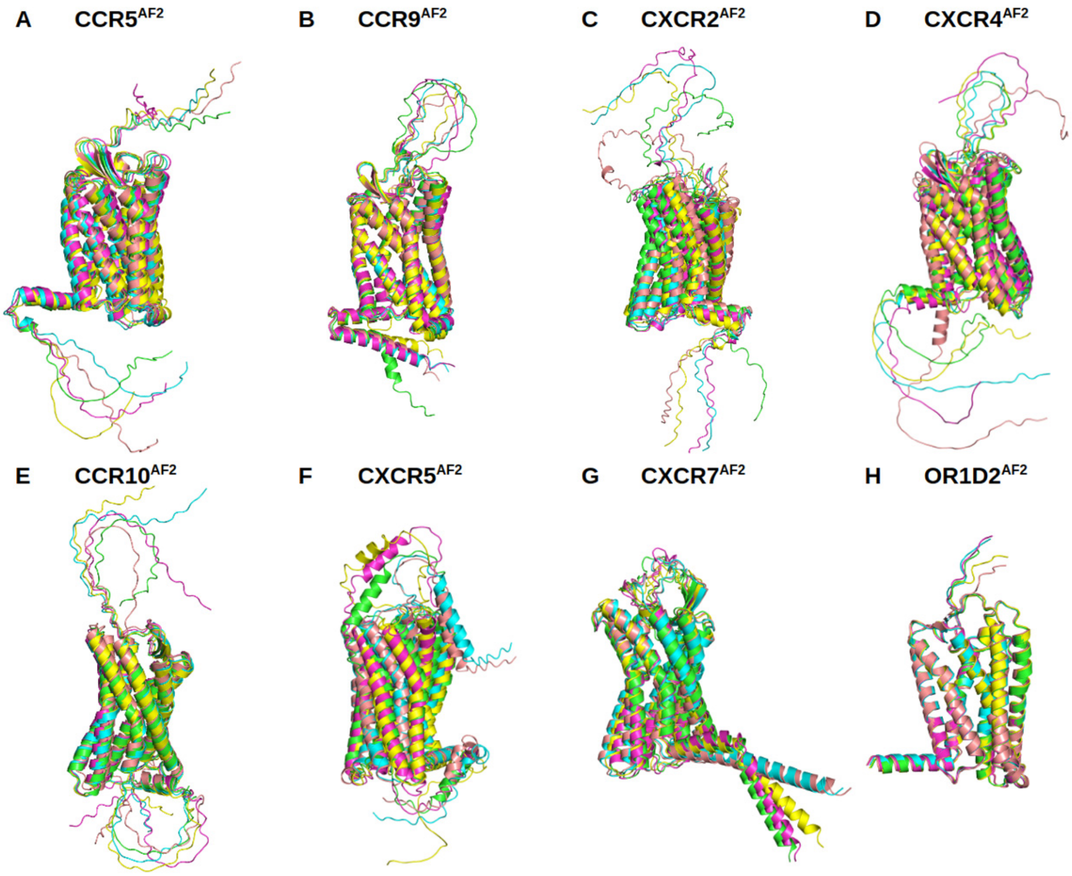

Figure S2. The AlphaFold2 predicted 5 structures of each GPCR. As seen from these superimposed structures, the variations are small especially for the transmembrane domains. The 5 colors represent 5 AlphaFold2 different predictions of the same protein sequence. .

All-atom model RMSD (see explanation below):
CCR5_M = [0.346, 0.281, 0.467, 0.925]
CCR9_M = [2.310, 2.475, 2.450, 2.228]
CCR10_M = [0.997, 0.892, 0.759, 0.937]
CXCR2_M = [0.836, 0.676, 0.773, 0.825]
CXCR4_M = [1.048, 0.966, 1.010, 4.672]
CXCR5_M = [9.067, 4.107, 3.431, 8.740]
CXCR7_M = [4.713, 0.815, 2.069, 4.967]
OR1D2_M = [0.524, 0.872, 0.499, 1.057]

Average RMSD:
CCR5 = 0.5048
CCR9 = 2.3658
CCR10 = 0.8962
CXCR2 = 0.7775
CXCR4 = 1.9240
CXCR5 = 6.3362
CXCR7 = 3.1410
OR1D2 = 0.7380

AF2 parameters:
homooligomer = 1
msa_method = mmseqs2
msa_format = fas
pair_mode = unpaired

pair_cov = 50
pair_qid = 20
rank_by = pLDDT
use_turbo = True
max_msa = 512:1024
show_images = True
num_models = 5
use_ptm = True
num_ensemble = 1
max_recycles = 3
tol = 0
num_samples = 1
subsample_msa = True
num_relax = None

Full-atom RMSD (no outlier rejection) and with superposition.

PyMOL command: align QTY and ss H, native and ss H, cycles=0, transform=1.

We superimpose only helices due to loops are not informative concerning the structure prediction, hence this RMSD is only based on helices atoms.

https://pymolwiki.org/index.php/Align

Figure S3. Five AlphaFold2 predicted models of CCR5$^{QTY.}$

a) Protein sequence of CCR5^{QTY}

MDYQVSSPIYDINYYTSEPCQKINVKQIAARLLPPQYSQTYTYGYTGNMQTTQTQTNCKRLKSMTDIYQQNQATSDQY
YQQTTPYWAHYAAAQWDFGNTMCQLLTGQYYTGYYSGTYYTTQQTTDRYLAVVHAVFALKARTVTYGTTTSTTTWT
TATYASQPGTTYTRSQKEGLHYTCSSHFPYSQYQFWKNFQTLKITTQGGQTQPQQTMTTCYSGTQKTQLRCRNEKKR
HRATRQTYTTMTTYYQYWAPYNTTQQLNTFQEFFGLNNCSSSNRLDQAMQVTETLGMTHCCINPIIYAFVGEKFRNYL
LVFFQKHIAKRFCKCCSIFQQEAPERASSVYTRSTGEQEISVGL

b) Sequence coverage
Total Length: 352

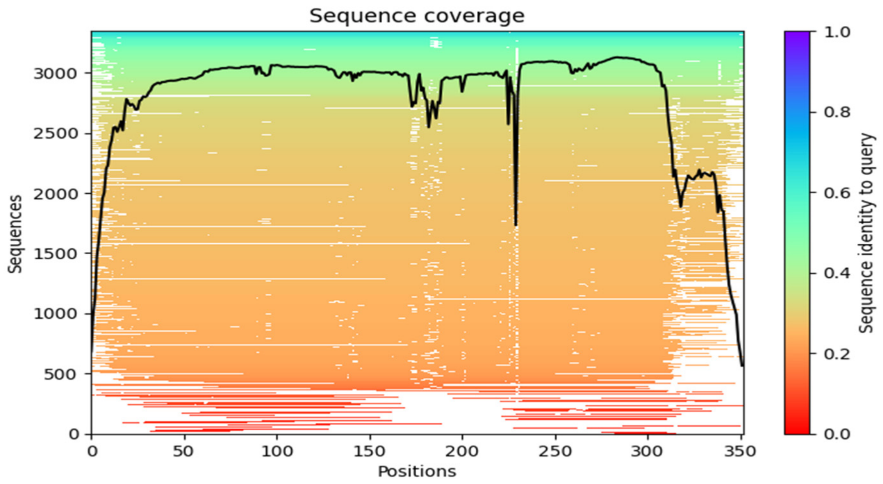

c) model_1_ptm_seed_0 recycles:3 tol:2.20 pLDDT:78.00 pTMscore:0.76

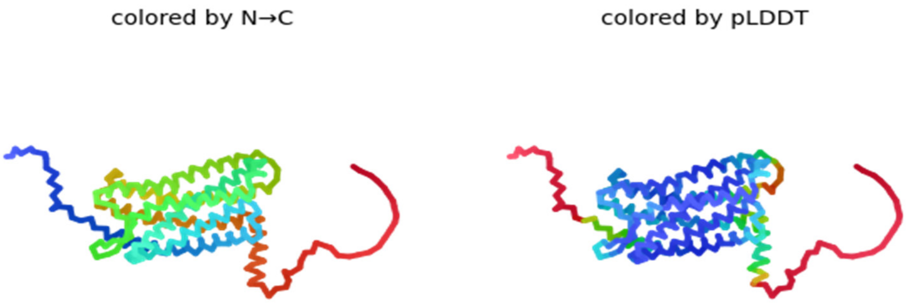

d) model_2_ptm_seed_0 recycles:3 tol:2.06 pLDDT:74.88 pTMscore:0.75

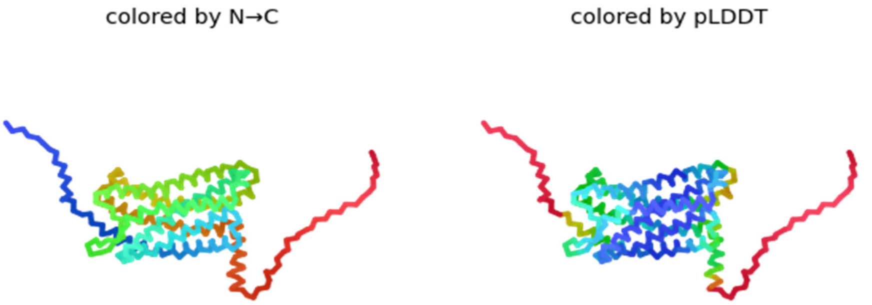

e) model_3_ptm_seed_0 recycles:3 tol:0.90 pLDDT:82.36 pTMscore:0.79

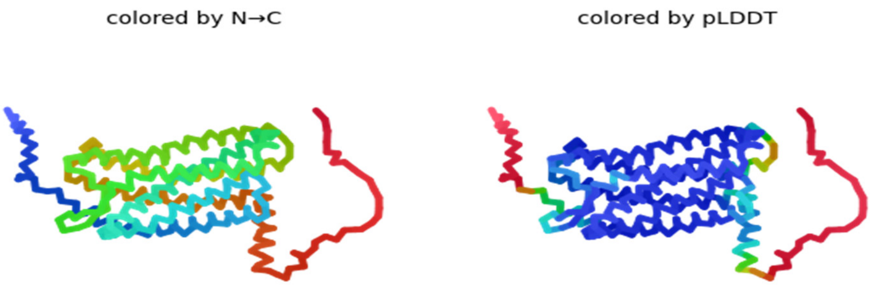

f) model_4_ptm_seed_0 recycles:3 tol:1.68 pLDDT:80.64 pTMscore:0.79

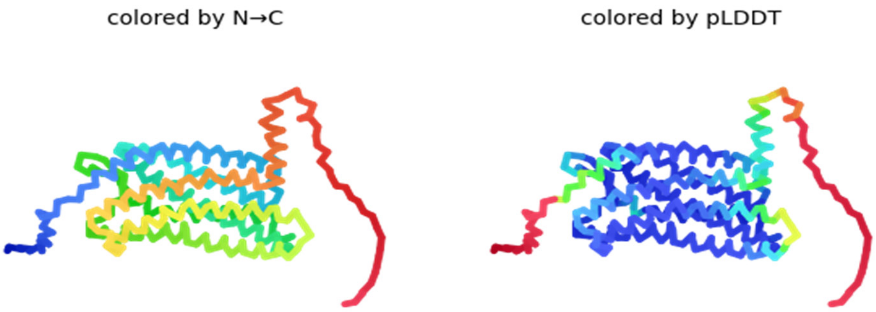

g) model_5_ptm_seed_0 recycles:3 tol:0.93 pLDDT:80.30 pTMscore:0.79

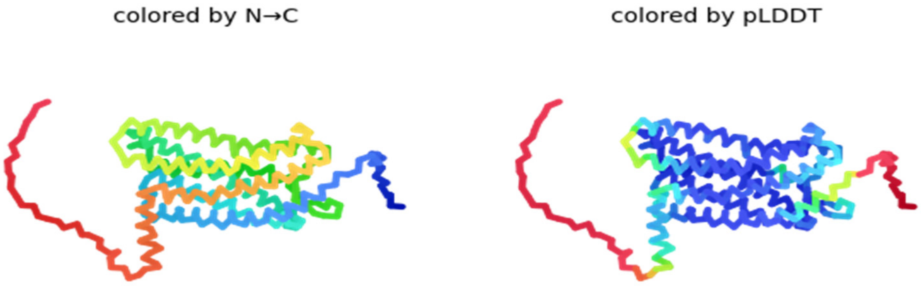

h) model rank based on pLDDT
rank_1_model_3_ptm_seed_0 pLDDT:82.36
rank_2_model_4_ptm_seed_0 pLDDT:80.64
rank_3_model_5_ptm_seed_0 pLDDT:80.30
rank_4_model_1_ptm_seed_0 pLDDT:78.00
rank_5_model_2_ptm_seed_0 pLDDT:74.88

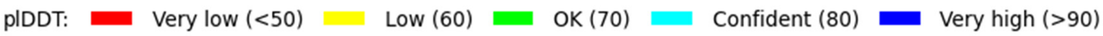

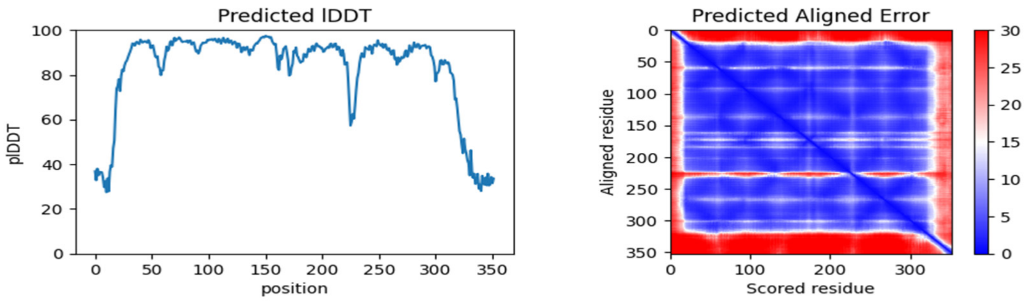

i) predicted alignment error

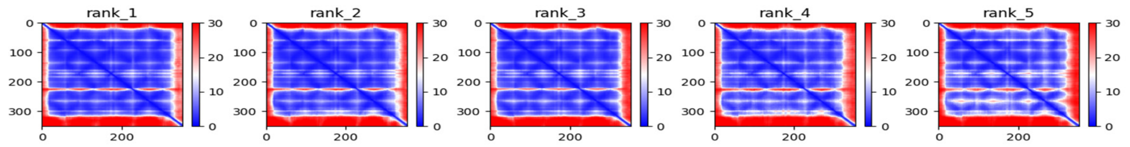

j) predicted contacts

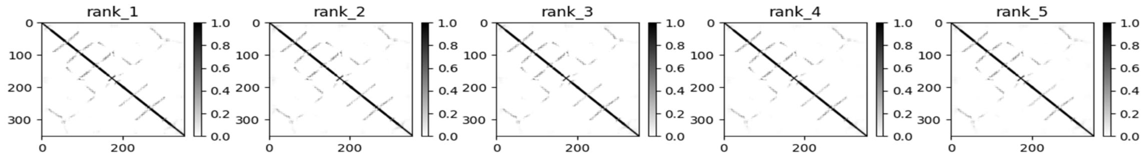

k) predicted distogram

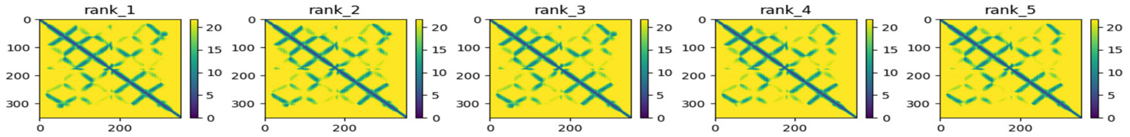

l) predicted LDDT

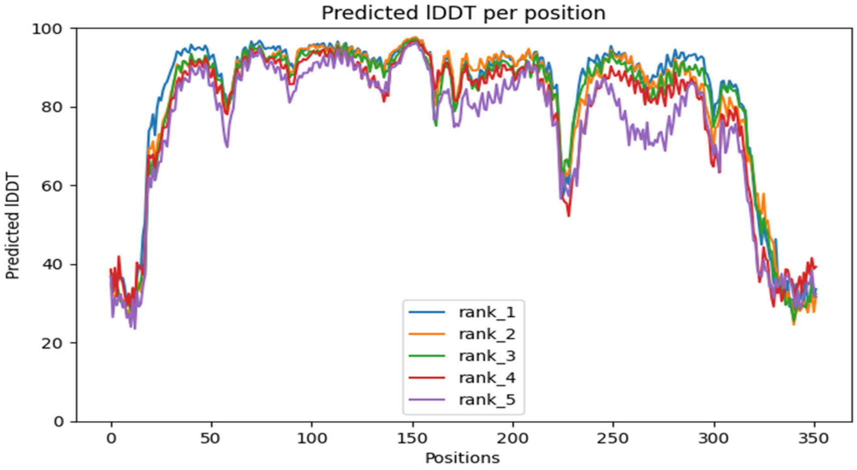

Supplement: Supplementary file 1 [file life-11-01285-s001.zip › life-1466984-supplementary.pdf]
